# Antagonistic effects of nano-copper on lead toxicity in Nile and blue Tilapia: Evidence from growth, immunity, and gene expression

**DOI:** 10.1016/j.cirep.2025.200258

**Published:** 2025-10-25

**Authors:** Mohammed F. El Basuini, Rawheya Shaaban Ramadan, Medhat E. Eldenary, Islam I. Teiba, Ali A. Soliman, Mahmoud S. Gewaily, Issam Khelfaoui, Mayada Alhoshy, Akram Ismael Shehata

**Affiliations:** a Faculty of Agriculture, Tanta University, Tanta 31527, Egypt; b King Salman International University, South Sinai 46618, Egypt; c Fish Nutrition Laboratory, Aquaculture Division, National Institute of Oceanography and Fisheries, Alexandria 21556, Egypt; d Department of Anatomy and Embryology, Faculty of Veterinary Medicine, Kafrelsheikh University, Kafr El-Sheikh 33516, Egypt; e School of Public Health, Shantou University/Institute of Local Government Development, Shantou University, Shantou 515063, China; f Independent Researcher, Alexandria city, Egypt; g Department of Animal and Fish Production, Faculty of Agriculture (Saba Basha), Alexandria University, Alexandria 21531, Egypt; h Institute of Marine Sciences, Shantou University, Shantou 515063, China

**Keywords:** Antagonistic effect, Antioxidant enzymes, Gene expression, Lead toxicity, Nano-copper, Tilapia

## Abstract

This study investigated the antagonistic effects of dietary nano-copper (Nano-Cu) against lead (Pb) toxicity in two tilapia species, *Oreochromis niloticus* (*n* = 360; 55.33 ± 0.54 g) and *Oreochromis aureus* (*n* = 360; 55.54 ± 0.61 g), over a 60-day feeding trial. The two tilapia species were divided into four dietary groups: control, Pb (100 µg/kg diet), Nano-Cu (2 mg/kg diet), and Pb+Nano-Cu. Growth performance, digestive enzyme activity, muscle Pb accumulation, serum biochemistry, immune responses, hepatic antioxidant status, and gene expression were assessed, along with histopathological examinations of the intestine and liver. Pb exposure significantly reduced growth indicators, digestive enzyme activities, antioxidant capacity, and immune function, while increasing hepatic malondialdehyde (MDA), serum glucose, liver enzymes, and pro-inflammatory gene expression. Histologically, Pb exposure caused evident structural damage, including villus atrophy in the intestine and hepatic vacuolation with sinusoidal dilatation. In contrast, Nano-Cu supplementation markedly improved growth, protein metabolism, enzymatic activities, and antioxidant status, and mitigated Pb accumulation and toxicity. Nano-Cu preserved normal intestinal and hepatic histoarchitecture and partially alleviated the Pb-induced tissue damage when co-administered. No significant species-specific differences or treatment **×** species interactions were observed for most variables. These findings demonstrate that Nano-Cu supplementation effectively counteracts Pb-induced toxicity in tilapia and enhances fish health by improving physiological function and maintaining tissue integrity. Future research should expand immunological profiling to include specific immune proteins (e.g., complement C3, IgM) and cytokines (e.g., TNF-α, IL-10), providing deeper mechanistic insight into Nano-Cu-mediated immune modulation under toxic stress, and further investigate its long-term effects under various environmental conditions.

## Highlights

• Dietary nano-copper mitigated lead (Pb) toxicity in *Oreochromis niloticus* and *O. aureus*.
• Nano-copper (2 mg/kg diet) improved growth, immunity, and antioxidant status in Pb-exposed fish.
• Pb increased hepatic MDA, inflammatory markers, and histopathological alterations.
• Nano-copper reduced tissue Pb accumulation and preserved intestinal and hepatic structure.
• Pb altered *IGF-1, CAT, IL-1β*, and *hepcidin* expression; Nano-Cu reversed these effects.
• No significant treatment × species interactions were observed across most parameters.

## Introduction

Copper (Cu) is an indispensable trace element in vertebrates, including teleost fish, owing to its involvement in numerous physiological and biochemical processes [1,2]. It functions as a catalytic cofactor in several vital enzymes such as superoxide dismutase (SOD), cytochrome c oxidase, tyrosinase, and dopamine β-hydroxylase, thereby facilitating redox regulation, mitochondrial respiration, neurotransmitter synthesis, and collagen and hemoglobin production [[3], [4], [5]]. Although Cu can be absorbed through both dietary and aqueous routes, dietary intake supplies the majority of total body Cu content in fish, far exceeding waterborne uptake [6]. As conventional feed ingredients often lack adequate levels of bioavailable Cu, exogenous supplementation becomes necessary to fulfill the metabolic demands of aquaculture species [7].

Both Cu deficiency and excess can result in physiological disturbances. Inadequate Cu levels have been linked to impaired enzymatic activities, compromised immune function, and growth retardation in several fish species [8,9]. On the contrary, overexposure to Cu, especially in ionic or poorly absorbed forms, can generate reactive oxygen species (ROS) that cause oxidative damage to lipids, proteins, and nucleic acids, thereby compromising cellular integrity and organ function [10,11]. Thus, the optimization of Cu supplementation strategies in aquaculture diets is essential to enhance growth, immunity, and oxidative balance, while minimizing toxicity risks and environmental pollution [7,12].

In recent years, nanotechnology has introduced novel mineral forms with enhanced functionality [13]. Nano-copper (Nano-Cu), owing to its high surface-to-volume ratio, improved solubility, and superior cellular absorption via endocytotic pathways, has emerged as a potent alternative to conventional Cu salts [14,15]. Studies have reported that Nano-Cu exerts beneficial effects in fish, including improved growth performance, immune response, and antioxidative enzyme activity, as well as reduced feed conversion ratios [2,16]. It enhances the cellular defense system by facilitating the synthesis and activity of Cu–Zn-superoxide dismutase, catalase (CAT), glutathione peroxidase (GPx), and glutathione-S-transferase (GST), which collectively mitigate oxidative stress and maintain redox homeostasis [1,3]. Additionally, Cu is involved in modulating the kynurenine pathway, which governs tryptophan catabolism; dysregulation of this pathway can result in the accumulation of neurotoxic intermediates, implicating Cu in neurological health [17,18].

The immunomodulatory effects of Cu are equally critical [19,20]. Cu supplementation has been shown to upregulate immune components such as ceruloplasmin, interleukin production, and the activity of splenocytes and phagocytes [[21], [22], [23]]. Furthermore, Nano-Cu contributes to stress resistance by supporting the expression of heat shock proteins and antioxidant vitamins such as ascorbic acid [1,24]. These diverse functions highlight the potential of Nano-Cu to not only enhance fish performance not only under normal conditions but also under environmental stressors.

Lead (Pb), a common aquatic contaminant, poses significant ecological and toxicological risks in aquaculture systems [25]. Chronic Pb exposure is known to interfere with multiple physiological systems, including the nervous, immune, and hematopoietic systems, often mediated through the induction of oxidative stress and inhibition of antioxidant defenses [26]. Pb toxicity in aquatic animals is associated with impaired growth, histopathological alterations, reduced immune competence, and changes in gene expression related to stress and detoxification [[27], [28], [29]]. While chelating agents and antioxidants have been investigated as mitigative strategies, little is known about how dietary nano-copper (Nano-Cu) modulates Pb toxicity across different tilapia species, a knowledge gap this study aims to address. Understanding species-specific physiological and molecular responses to Nano-Cu under Pb stress is essential for developing targeted nutritional strategies to improve resilience and productivity in aquaculture.

Tilapia species, particularly *Oreochromis niloticus* (Nile tilapia) and *Oreochromis aureus* (blue tilapia), are among the most widely cultured freshwater finfish globally due to their fast growth, tolerance to variable environmental conditions, and high market demand [30,31]. *O. niloticus* is the most dominant species in global tilapia aquaculture, characterized by rapid growth, high feed efficiency, and robust adaptability to a range of salinities and temperatures [32]. *O. aureus*, while slightly less prevalent, is noted for its greater cold tolerance, disease resistance, and reproductive control in mixed populations [33]. Both species play essential roles in food security and the aquaculture economy, particularly in low- and middle-income countries [34]. However, their intensive production systems often expose them to multiple stressors, including heavy metals such as lead [27,35].

In this context, the present study specifically addresses the limited understanding of the antagonistic interactions between nano-copper (Nano-Cu) and lead (Pb) toxicity in tilapia, with a focus on comparative species responses between *O. niloticus* and *O. aureus*. While numerous studies have described either the beneficial effects of Nano-Cu or the harmful consequences of Pb exposure independently, no prior research has systematically evaluated their interactive effects within and across tilapia species. This investigation therefore aims to clarify whether Nano-Cu supplementation can counteract Pb-induced physiological, immunological, and molecular disturbances, thereby contributing to the development of targeted nutritional interventions for metal-stressed aquaculture systems.

## Materials and methods

### Ethics, experimental fish, acclimatization, and laboratory conditions

A total of 720 healthy juvenile tilapia, comprising *Oreochromis niloticus* (*n* = 360; average initial weight = 55.33 ± 0.54 g) and *Oreochromis aureus* (*n* = 360; average initial weight = 55.54 ± 0.61 g), were procured from a commercial fish hatchery in Kafr Elsheikh City, Egypt. Prior to the commencement of the experiment, all fish were transported in oxygenated tanks to the Baltim Research Station, National Institute of Oceanography and Fisheries (NIOF), Egypt. Upon arrival, fish were acclimatized for two weeks in fiberglass tanks (1000 L capacity) under optimal environmental conditions (temperature: 26 ± 1 °C; dissolved oxygen: >5 mg/L; pH: 7.5 ± 0.2). During acclimation, fish were fed a basal diet devoid of Nano-Cu or Pb. This study fully adheres to the ARRIVE guidelines version 2.0 to ensure transparent and ethical reporting of animal research. Ethical approval was granted by the Animal Care and Use Committee of the Desert Agriculture College, King Salman International University, Egypt (Approval No. KSIU/2025/DA-8). All procedures complied with national and institutional regulations, and efforts were made to minimize animal suffering, in accordance with the principles of the 3Rs (Replacement, Reduction, and Refinement).

### Experimental design, dietary treatments, and diet preparation

After acclimatization, fish from each species were randomly allocated into four dietary treatment groups, with three replicate tanks per treatment (30 fish per replicate). The treatment groups were designed to evaluate the independent and combined effects of dietary nano-copper and lead. The first group served as the control, receiving a basal diet with no supplementation. The second group was fed the basal diet supplemented with lead (Pb) at a concentration of 100 µg/kg, administered in the form of lead acetate. The third group received a basal diet enriched with nano-copper (Nano-Cu) at a level of 2 mg/kg, using elemental nano-Cu particles. The fourth group was given a diet containing a combination of both Pb (100 µg/kg) and nano-Cu (2 mg/kg) to assess the antagonistic effect of nano-copper on lead-induced toxicity.

The feeding trial was conducted over a 60-day period. Fish were hand-fed to apparent satiation twice daily (08:00 and 16:00). Uneaten feed and fecal residues were removed daily by siphoning, and approximately 30 % of the water in each tank was exchanged to maintain optimal water quality throughout the trial (temperature: 26.7 ± 0.54 °C; dissolved oxygen: >5.77 mg/L; pH: 7.6 ± 0.7; 12-h light/dark cycle). Diets were isonitrogenous (29.53 ± 0.24 % crude protein) and isolipidic (8.62 ± 0.19 % crude fat) [36] (Table 1). The base feed ingredients were finely ground, homogeneously mixed, and pelletized through a 2-mm die using a laboratory-scale pellet mill. The pelleted feeds were dried at 45 °C and stored at 4 °C until feeding. Nano-copper (Nano-Cu) with 99 % purity and a particle size less than 75 nm was obtained from Sigma-Aldrich (Catalog No. 207,780–500 G, USA). The material identity and size range were confirmed according to the manufacturer’s certificate of analysis. The supplier specifies metallic elemental copper nanoparticles without polymeric or oxide coatings. Prior to incorporation into the feed, Nano-Cu was dispersed in deionized water and probe-sonicated (100 W, 40 % amplitude, 3 × 5 min with cooling intervals) to enhance homogeneity and reduce aggregation. The inclusion level (2 mg/kg diet) followed the established and biologically validated dose reported by El Basuini, et al. [16]. Lead was incorporated into the diet as lead acetate (analytical grade, Sigma-Aldrich) at a concentration of 100 µg/kg [29,37]. Both nano-copper and lead acetate were carefully blended into the feed matrix to ensure homogenous distribution Table 1.

**Table 1:** Basal Diet Composition and Nutritional Content ( %, Dry Matter Basis; *n* = 3).

Ingredient	Inclusion (%)	Chemical profile	%
Fish meal (72 %)	10.5	Crude protein	29.53 ± 0.24
Soybean meal (44 %)	42	Crude lipid	8.62 ± 0.19
Wheat bran	10	Crude fiber	5.36 ± 0.11
Yellow corn	18.5	Ash	4.74 ± 0.14
Rice bran	10		
Starch	3		
Fish oil	2.5		
Sunflower oil	1.5		
Premix ^a^	2		
**Total**	**100**		

^a^ The composition of the premix is described in detail by Shehata et al. [36].

### Growth performance and survival rate

At the start and end of the 60-day feeding trial, individual body weights of *Oreochromis niloticus* and *O. aureus* were recorded to assess growth performance. Weight gain (WG %), specific growth rate (SGR), feed conversion ratio (FCR), and survival rate (SR %) were calculated using standard equations.

Weight gain (WG,%)

$$\text{WG}\;=\frac{W_{60\;}-\;W_{0\;}}{W_{0\;\text{day}}}\times \;100$$

Specific growth rate (SGR, %/day)

$$\text{SGR}=\frac{\text{Ln}\;W_{60}-\text{Ln}\;W_{O}}{60}\times \;100$$

Feed conversion ratio (FCR)

$$\text{FCR}=\frac{\text{Total}\;\text{feed}\;\text{intake}\;\left(g\right)}{\text{WG}\;\left(g\right)}$$

Survival rate (SR, %)

$$\text{SR}\;=\frac{\text{Final}\;\text{number}\;\text{of}\;\text{fish}}{\text{Initial}\;\text{number}\;\text{of}\;\text{fish}}\times \;100$$

### Sampling procedure

At the end of the trial (day 60), six fish per replicate (18 fish per treatment group) were randomly sampled and anesthetized with clove oil (50 mg/L) for tissue and blood collection. Blood samples were drawn from the caudal vein using sterile syringes and centrifuged (3000 rpm, 10 min) to obtain serum, which was stored at −80 °C for biochemical and immunological analyses. Liver and intestinal tissues were collected for antioxidant assays, gene expression analysis, and histopathological examination. Muscle samples were obtained to assess Pb accumulation.

### Digestive enzyme assays and lead residual determination

To evaluate digestive enzyme activities, intestinal tissues were harvested immediately post-dissection. Each sample was rinsed with ice-cold phosphate-buffered saline (PBS, pH 7.5; 1:10 w/v), homogenized on ice, and centrifuged at 5000 × *g* for 5 min at 4 °C. Protease activity was determined using a commercially available assay kit (Sigma-Aldrich, USA), employing casein as the substrate per the method of Cupp-Enyard [38]. Amylase and lipase activities were assessed spectrophotometrically at 714 nm and 540 nm, respectively, as described by Wang, et al. [39]. All enzymatic measurements were carried out under standardized laboratory conditions to ensure data reliability and reproducibility.

The analysis of lead (Pb) residues in fish tissue was conducted following the procedure outlined by Gebremedhin and Berhanu [40]. Briefly, 0.5 g of dried fish muscle samples were subjected to acid digestion for 5 h using a solution composed of 2.0 ml perchloric acid (70 %, Spectrosol), 6.0 ml nitric acid (70 %, Spectrosol), and 4.0 ml hydrogen peroxide (35 %, Riedel-de Haen). Once digestion was complete, the resulting solution was transferred to a 25 mL volumetric flask and brought to final volume with distilled water. Lead concentrations in the prepared samples were subsequently determined using flame atomic absorption spectrophotometry, with instrument calibration performed using standard lead solutions.

### Histomorphological examination

Upon completion of the 60-day feeding trial, liver and intestinal tissues were excised from representative fish specimens for histological analysis, following standardized protocols as outlined by Abumandour and Gewaily [41]. The collected samples were immediately fixed in 10 % neutral-buffered formalin to preserve tissue integrity. Thereafter, specimens were processed through a graded ethanol series (70 % to 100 %) for dehydration, cleared in xylene, and embedded in paraffin wax. Paraffin-embedded tissues were sectioned at a thickness of 5 µm using a rotary microtome (Leica RM 2035, Leica Microsystems, Germany). Sections were carefully mounted onto adhesive-coated glass slides and stained with hematoxylin and eosin (H&E) in accordance with the staining procedure described by Bancroft and Gamble [42]. Prepared slides were examined under a Leica DM500 light microscope, and digital images were captured using a Leica EC3 camera to document and evaluate histopathological alterations among the experimental groups.

### Serum biochemistry, antioxidant defense, and innate immune responses

To evaluate the physiological and immunological effects of dietary treatments, a comprehensive suite of serum biochemical, hepatic antioxidant, and innate immune parameters was analyzed at the end of the 60-day feeding trial. Biochemical markers, including glucose, total protein, albumin, calculated globulin (total protein minus albumin), total cholesterol, triglycerides, alanine aminotransferase (ALT), aspartate aminotransferase (AST), urea, and creatinine, were quantified using commercially available colorimetric diagnostic kits (Bio-Diagnostic, Egypt), by the manufacturer’s protocols. Cortisol levels were determined via enzyme-linked immunosorbent assay (ELISA) using a commercially sourced kit (Calbiotech, USA; Cat. No. CO368S).

For the evaluation of hepatic antioxidant defenses, liver tissues were aseptically excised and immediately homogenized in cold 0.86 % saline solution (1:9 w/v). The homogenates were centrifuged at 13,600 × *g* for 10 min at 4 °C, and the supernatants were collected for enzymatic analysis. All sample preparations were performed under aseptic conditions, with coded labeling to ensure blind analysis and minimize experimental bias. Antioxidant enzyme activities, superoxide dismutase (SOD; WST-1 method, 450 nm), catalase (CAT; 405 nm), and glutathione peroxidase (GPx; 412 nm), were quantified using assay kits provided by Nanjing Jiancheng Bioengineering Institute (China). Lipid peroxidation was assessed by measuring malondialdehyde (MDA) content at 532 nm, serving as an index of oxidative damage. Innate immune responses were assessed through the quantification of lysozyme activity using the turbidimetric assay, bactericidal activity against *Streptococus agalactiae*, and neutrophil respiratory burst activity employing the nitroblue tetrazolium (NBT) reduction method [43].

### RNA isolation and quantitative real-time PCR analysis

Liver tissues designated for transcriptomic analysis were preserved in RNAlater® solution (Sigma-Aldrich, USA) at 4 °C for 24 h, then stored at −80 °C until RNA extraction. Total RNA was extracted using the A.B.T.™ RNA Purification Kit (Applied Biotechnology, Egypt), following the manufacturer’s guidelines. RNA concentration and purity were assessed using a NanoDrop spectrophotometer (BioDrop, UK), and samples were normalized to a final working concentration of 50 ng/μL. The RNA purity ranged between 1.9–2.0 (A260/280) and 2.0–2.2 (A260/230), indicating high purity. Gene-specific primers (listed in Table 2) were designed using Primer 5.0 software and validated for use in quantitative expression studies. Reverse transcription and quantitative PCR (RT-qPCR) were conducted in a one-step format utilizing SYBR Green-based detection chemistry. The thermal protocol comprised an initial reverse transcription step at 50 °C for 30 min, followed by denaturation at 95 °C for 10 min, and 45 amplification cycles of 95 °C for 5 s and 60 °C for 30 s for annealing and extension. The housekeeping gene *elongation factor 1-alpha* (*EF-1a*) was employed as an internal control to normalize gene expression levels. Melt curve analysis was performed to verify the specificity of amplification. Relative mRNA expression was determined using the 2^−ΔΔCt^ method [44].

**Table 2:** Quantitative real-time PCR (RT-qPCR) primer sequences.

Gene	Sequences (5′−3′)	Amplicaon size (bp)	Annealing (°C)	Efficiency%	Accession No.
*EF-1a*	F: GCACGCTCTGCTGGCCTTT	250	60	98	**KJ123689.1**
	R: GCGCTCAATCTTCCATCCC				
*IGF*-1	F: GTGGACGAGTGCTGCTTC	139	58	98	**XM_019346352.2**
	R: TGCTACTAACCTTGGGTGC				
*CAT*	F: GGCCGGGTTTCTAAAAGAAG	150	58	96	**XM_003447521.5**
	R: TAAACGTGCAAAGTGGCATC				
*IL-1β*	F: TGCTGAGCACAGAATTCCAG	172	60	99	**XM_019365841.2**
	R: GCTGTGGAGAAGAACCAAGC				
*Hep*	F: ACACTCGTGCTCGCCTTTAT	173	56	97	**MH651359.1**
	R: AGATGGCTCTGACGCTTTTG				

*EF-1a*: elongation factor 1-alpha (houskeeping gene); *IGF-1*: insulin-like Growth Factor 1; *CAT*: catalase; *IL-1 β*: interleukin-1 Beta; *Hep*: hepcidin.

### Data collection and statistical analysis

All experimental data, including growth performance, survival rate, biochemical and antioxidant markers, digestive enzyme activity, immune responses, and gene expression, were expressed as mean ± standard error (SEM). Data normality and homogeneity of variances were confirmed using Shapiro–Wilk and Levene’s tests. Two-way ANOVA was used to evaluate the effects of species, dietary treatments, and their interaction, followed by Tukey’s post hoc test for multiple comparisons when significant differences were observed (*p* < 0.05). Statistical analyses were performed using SPSS software (version 25.0; IBM Corp., USA).

## Results

### Performance parameters

Table 3 illustrates the effects of dietary nano-copper (Nano-Cu), lead (Pb), and their combination on growth performance and survival rate in *Oreochromis niloticus* and *Oreochromis aureus* after a 60-day feeding trial. The treatment factor showed a highly significant impact (*p* < 0.001) on final body weight (BW_60_), weight gain % (WG %), and specific growth rate (SGR), while also significantly affecting feed conversion ratio (FCR) (*p* = 0.001). In contrast, survival rate (SR) was not significantly affected by treatment, species, or their interaction (*p* > 0.05). Within each species, dietary lead markedly reduced BW_60_, WG %, and SGR compared to the control group. However, supplementation with Nano-Cu alone significantly enhanced performance parameters in both species, as evidenced by the highest BW_60_ (137.70 g in *O. niloticus* and 139.57 g in *O. aureus*), WG % (149.06 % and 150.58 %), and SGR (1.52 and 1.53 %/day), with improved FCR values (1.69 and 1.71, respectively). Interestingly, the co-supplementation of Pb and Nano-Cu partially alleviated the Pb-induced growth suppression, as growth metrics were intermediate between the Pb group and Nano-Cu group.

**Table 3:** Evaluation of performance parameters and survival rate in tilapias after a 60-day feeding trial.

Species	Treatments	BW_0_^1^	BW_60_^2^	WG % 3	SGR 4	FCR 5	SR 6
*Oreochromis niloticus*	Control	55.40	121.73 ^B^	119.8 ^B^	1.31 ^B^	1.87 ^B^	96.67
	Pb	55.38	105.88 ^C^	91.17 ^C^	1.08 ^C^	2.29 ^A^	96.67
	Nano-Cu	55.29	137.7 ^A^	149.06 ^A^	1.52 ^A^	1.69 ^B^	98.89
	Pb+Nano-Cu	55.24	117.9 ^B^	113.44 ^B^	1.26 ^B^	2 ^AB^	97.78
*Oreochromis aureus*	Control	55.35	126.96 ^b^	129.36 ^b^	1.38 ^b^	1.87 ^ab^	96.67
	Pb	55.37	115.66 ^c^	108.98 ^c^	1.23 ^c^	1.97 ^a^	96.67
	Nano-Cu	55.72	139.57 ^a^	150.58 ^a^	1.53 ^a^	1.71 ^b^	100
	Pb+Nano-Cu	55.72	127.45 ^b^	128.77 ^b^	1.38 ^b^	1.9 ^a^	98.89
S.E.M.		0.4	2.43	4.97	0.04	0.08	1.67
*P*-Value	Species	0.462	0.001	0.006	0.005	0.105	0.644
	Treatment	0.98	0.000	0.000	0.000	0.001	0.299
	Interaction	0.861	0.336	0.392	0.332	0.186	0.973

1 Body weight at 0-day.

2 Body weight at 60-day.

3 Weight gain, %

4 Specific growth rate, %/day.

5 Feed conversion ratio.

6 Survival rate, %. Different letters indicate significant differences within each species at *P* < 0.05 (uppercase letters ^A,B,C,…etc.^: *Oreochromis niloticus*; lowercase letters ^a,b,c,…etc.^: *Oreochromis aureus*).

### Digestive enzymes

Table 4 displays the influence of dietary nano-copper (Nano-Cu), lead (Pb), and their combination on digestive enzyme activities (amylase, lipase, and protease) in *Oreochromis niloticus* and *Oreochromis aureus* after 60 days of feeding. A highly significant effect of treatment was observed on all enzyme activities (*p* < 0.005), with species-specific differences detected only for lipase activity (*p* < 0.001), and a significant species × treatment interaction for lipase as well (*p* = 0.008). In terms of amylase activity, fish fed the Nano-Cu-supplemented diet exhibited significantly higher values in both species compared to the Pb group, which recorded the lowest enzyme levels. Notably, *O. niloticus* and *O. aureus* treated with Nano-Cu showed amylase values of 20.59 and 20.83 U/mg protein, respectively, whereas Pb exposure reduced amylase to 16.44 and 17.27 U/mg. A similar pattern was observed for protease activity, where Nano-Cu enhanced protease levels significantly (21.40 in *O. niloticus* and 21.67 in *O. aureus*), while Pb led to marked reductions, especially in *O. niloticus* (16.64 U/mg). Regarding lipase activity, species differences were pronounced, with *O. aureus* generally exhibiting higher values than *O. niloticus* under control and Nano-Cu treatments. For instance, lipase activity in *O. aureus* reached 24.19 U/mg with Nano-Cu, compared to 23.69 in *O. niloticus*, while the Pb group showed decreased levels (21.27 and 19.03, respectively). The interaction effect was also evident in the lipase data, where Nano-Cu reversed Pb-induced suppression more effectively in *O. aureus* than in *O. niloticus*.

**Table 4:** Digestive enzyme activities of tilapias after a 60-day feeding trial.

Species	Treatments	Amylase	Lipase	Protease
*Oreochromis niloticus*	Control	18.70 ^AB^	19.32 ^B^	19.05 ^B^
	Pb	16.44 ^B^	19.03 ^B^	16.64 ^C^
	Nano-Cu	20.59 ^A^	23.69 ^A^	21.40 ^A^
	Pb+Nano-Cu	18.64 ^AB^	20.44 ^B^	18.98 ^B^
*Oreochromis aureus*	Control	18.95 ^ab^	23.95 ^a^	19.24 ^a^
	Pb	17.27 ^b^	21.27 ^b^	18.76 ^a^
	Nano-Cu	20.83 ^a^	24.19 ^a^	21.67 ^a^
	Pb+Nano-Cu	18.74 ^ab^	21.70 ^b^	20.65 ^a^
S.E.M.		0.78	0.53	0.87
*P*-Value	Species	0.530	0.000	0.102
	Treatment	0.002	0.000	0.004
	Interaction	0.967	0.008	0.598

Different letters indicate significant differences within each species at *P* < 0.05 (uppercase letters ^A,B,C,…etc.^: *Oreochromis niloticus*; lowercase letters ^a,b,c,…etc.^: *Oreochromis aureus*).

### Histological assessment and lead accumulation

**Fig. 1:**
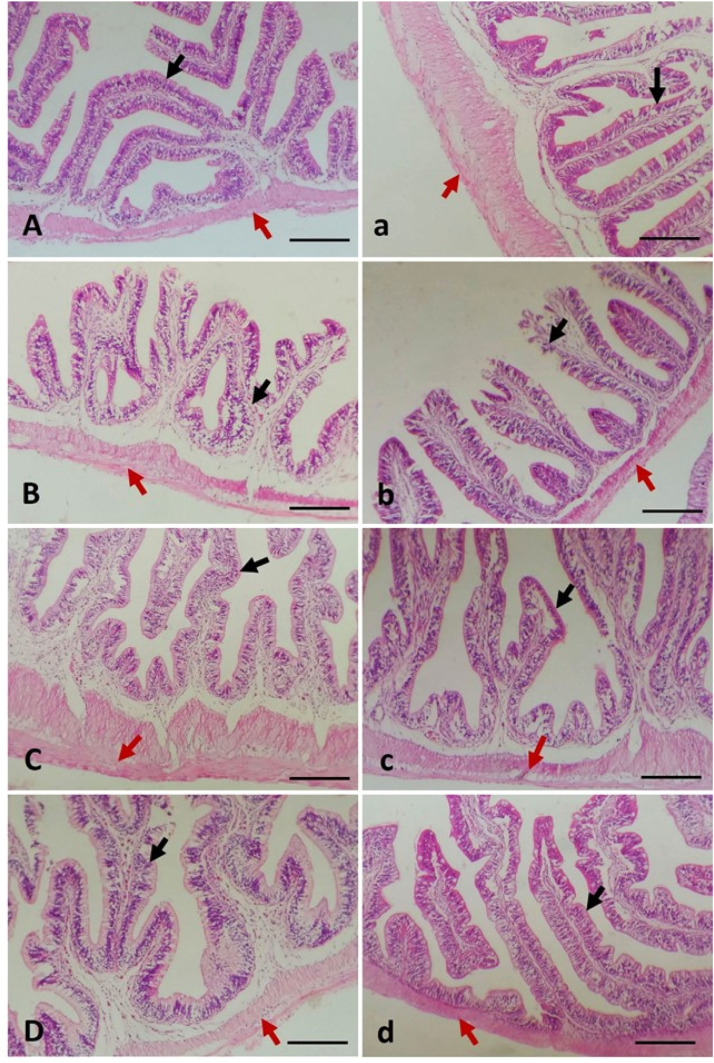
Photomicrographs of the intestines of *Oreochromis niloticus* (A–D) and *Oreochromis aureus* (a–d). The first group in each species (A, a) served as the control and was fed a basal diet without any additives. The second group (B, b) received the basal diet contaminated with 100 µg of lead (Pb). The third group (C, c) was fed the basal diet supplemented with 2 mg of copper nanoparticles (Nano-Cu). The fourth group (D, d) received a diet contaminated with 100 µg of Pb and supplemented with 2 mg of Nano-Cu. The intestinal villi (black arrow) and the intestinal wall (red arrow). H&E stain. Bar = 100 µm.

**Fig. 2:**
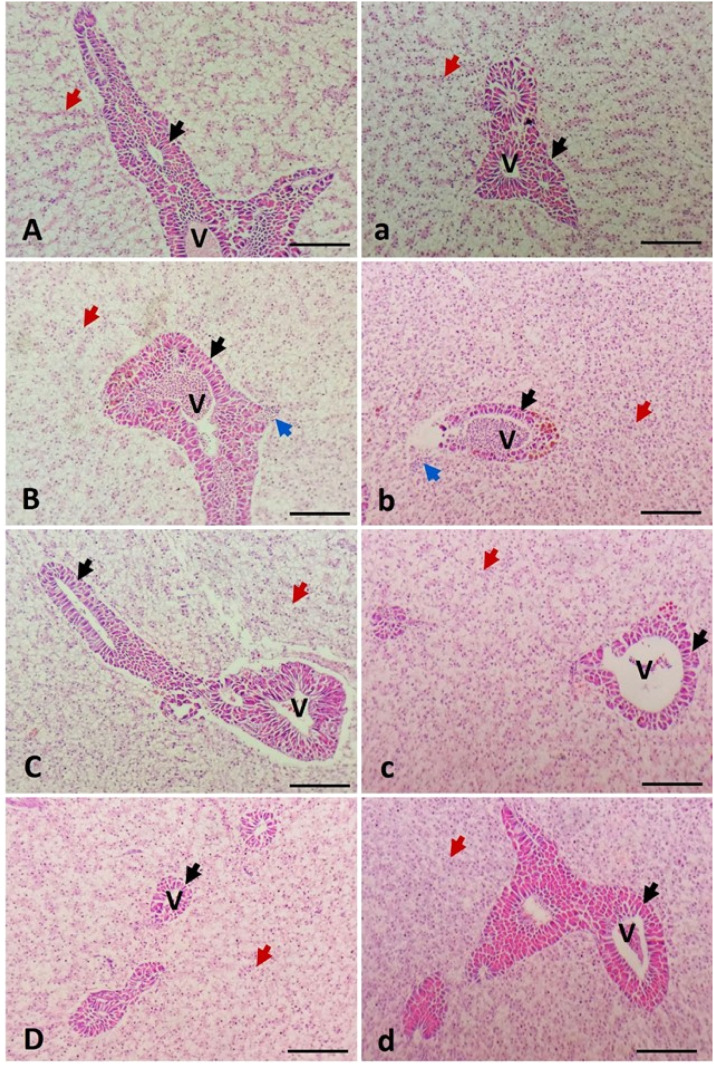
Photomicrographs of the livers of *Oreochromis niloticus* (A–D) and *Oreochromis aureus* (a–d). The first group in each species (A, a) served as the control and was fed a basal diet without any additives. The second group (B, b) received the basal diet contaminated with 100 µg of lead (Pb). The third group (C, c) was fed the basal diet supplemented with 2 mg of copper nanoparticles (Nano-Cu). The fourth group (D, d) received a diet contaminated with 100 µg of Pb and supplemented with 2 mg of Nano-Cu. The hepatocytes (red arrow), the pancreatic acini (black arrow), the central vein (V), and inflammatory cell infiltration (blue arrow). H&E stain. Bar = 100 µm.

Figs. 1 and 2 collectively illustrate the histopathological responses in the intestines and livers, respectively, of *Oreochromis niloticus* and *Oreochromis aureus* following a 60-day dietary exposure to lead (Pb), nano-copper (Nano-Cu), and their combination.

Fig. 1 presents the intestinal architecture of both tilapia species across all treatment groups. In the control group (A, a), the intestinal mucosa appeared intact with well-defined villi, uniform epithelial lining, and no evident pathological alterations. Exposure to dietary Pb alone (B, b) induced significant structural damage, including shortened and blunted villi, epithelial sloughing, and mucosal disorganization. Conversely, fish fed Nano-Cu alone (C, c) showed preserved villus morphology, with mild enhancement in tissue integrity compared to control. Notably, the group exposed to both Pb and Nano-Cu (D, d) exhibited marked histological improvement over the Pb-only group, with partial restoration of villus height and mucosal architecture, suggesting an ameliorative effect of Nano-Cu against Pb-induced intestinal damage.

Similarly, Fig. 2 demonstrates the liver histology of the experimental groups. In both *O. niloticus* and *O. aureus*, the control livers (A, a) displayed normal hepatic parenchyma with regularly arranged hepatocytes and well-preserved pancreatic acini. Pb-exposed fish (B, b) showed hepatic degeneration, sinusoidal dilatation, and inflammatory infiltration, indicating pronounced toxic effects. In contrast, the Nano-Cu group (C, c) retained near-normal histological features with slight vacuolization, reflecting the biocompatibility of Nano-Cu at the administered dose. The co-exposed group (D, d) demonstrated substantial histopathological recovery, with improved hepatic architecture and reduced lesions compared to the Pb group, further supporting the protective role of Nano-Cu in counteracting Pb toxicity.

**Fig. 3:**
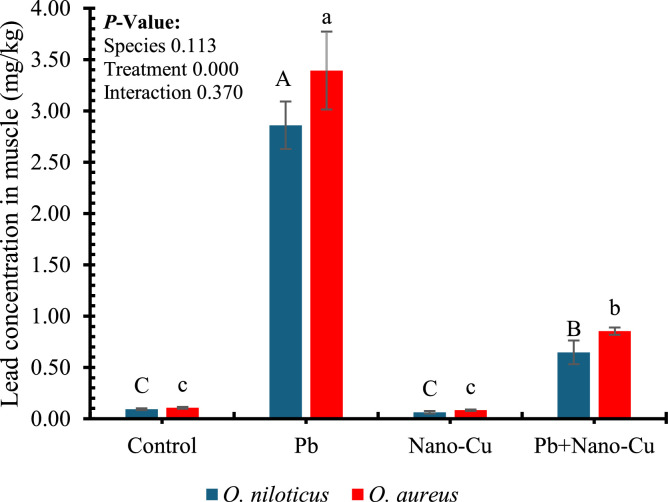
Lead concentration in the muscle of tilapias after 60 days of feeding trial. Different letters indicate significant differences within each species at *P* < 0.05 (uppercase letters ^A, B, C,…etc.^: *Oreochromis niloticus*; lowercase letters ^a, b, c,…etc.^: *Oreochromis aureus*).

Fig. 3 demonstrates the effect of dietary lead (Pb), nano-copper (Nano-Cu), and their combination on muscle lead accumulation in *Oreochromis niloticus* and *Oreochromis aureus* after 60 days of feeding. A highly significant treatment effect was observed (*p* < 0.001), while neither species nor the interaction between species and treatment showed statistically significant effects (*p* > 0.05). Fish exposed to Pb alone exhibited a marked increase in muscle lead concentration, reaching the highest values in both species (approximately 2.8 mg/kg in *O. niloticus* and 3.4 mg/kg in *O. aureus*), significantly exceeding all other treatments. In contrast, fish in the control and Nano-Cu groups showed minimal lead accumulation, with values remaining below 0.2 mg/kg and statistically similar within species (*p* < 0.05). Importantly, co-supplementation with Nano-Cu significantly reduced the Pb burden in muscle tissue compared to the Pb-only group, though values remained higher than control. For instance, Pb+Nano-Cu fish showed intermediate lead levels (around 1.0–1.2 mg/kg), indicating a partial ameliorative effect of Nano-Cu against Pb bioaccumulation.

### Serum biochemical parameters

Table 5 presents the effects of dietary lead (Pb), nano-copper (Nano-Cu), and their combination on the serum biochemical parameters of *Oreochromis niloticus* and *Oreochromis aureus* after a 60-day feeding trial. Among the measured indices, treatment exerted a statistically significant effect on total protein (TP; *p* = 0.0005), globulin (GLOB; *p* = 0.0003), glucose (GLUC; *p* < 0.0001), total cholesterol (TChol; *p* < 0.0001), and alanine aminotransferase (ALT; *p* = 0.0064), whereas species and interaction effects remained non-significant (*p* > 0.05 for all). In both species, Pb exposure significantly elevated serum glucose levels (105.33 mg/dL in *O. niloticus* and 109.33 mg/dL in *O. aureus*) compared to other treatments, indicating a stress-induced hyperglycemic response. Meanwhile, Nano-Cu alone led to a marked reduction in glucose levels (86.33 and 91.00 mg/dL, respectively), with intermediate values observed in the Pb+Nano-Cu group, reflecting a partial mitigation of Pb-induced hyperglycemia. Regarding protein profile, Nano-Cu supplementation significantly enhanced TP and globulin concentrations compared to control and Pb-treated fish, particularly in *O. niloticus*, where TP reached 4.31–4.37 g/dL and GLOB exceeded 2.8 g/dL. Conversely, Pb exposure did not significantly alter albumin levels but was associated with lower TP and globulin, reflecting impaired protein synthesis or immune suppression. For lipid metabolism, TChol levels were significantly decreased in Nano-Cu and Pb+Nano-Cu groups across both species, with the lowest values observed in *O. niloticus* (≈11 mg/dL) compared to higher values in control and Pb treatments (≈15–16 mg/dL), indicating Nano-Cu’s hypocholesterolemic potential. In terms of liver function enzymes, Pb exposure significantly elevated ALT activity (7.75 U/L in *O. niloticus*), while Nano-Cu maintained values closer to control levels. No significant treatment effect was observed for AST, triglycerides, urea, or creatinine levels.

**Table 5:** Serum biochemical indices of tilapias following 60 days of feeding.

Species	Treatments	TP 1	ALB 2	GLOB 3	GLUC 4	TChol 5	Trig 6	ALT 7	AST 8	Urea	Creatinine
*Oreochromis niloticus*	Control	3.43 ^B^	1.49	1.93 ^B^	96.67 ^B^	15.91 ^A^	62.00	5.52 ^B^	59.33	5.24	0.39
	Pb	3.44 ^B^	1.46	1.98 ^B^	105.33 ^A^	15.24 ^A^	63.00	7.75 ^A^	64.67	5.24	0.40
	Nano-Cu	4.31 ^A^	1.50	2.82 ^A^	86.33 ^C^	11.01 ^B^	62.00	5.61 ^B^	58.33	5.27	0.39
	Pb+Nano-Cu	4.37 ^A^	1.51	2.85 ^A^	99.33 ^B^	10.85 ^B^	62.33	6.09 ^AB^	59.67	5.30	0.41
*Oreochromis aureus*	Control	3.51	1.52	1.99 ^b^	97.33 ^bc^	15.55 ^a^	61.67	5.61	59.67	5.19	0.42
	Pb	3.63	1.48	2.15 ^b^	109.33 ^a^	16.27 ^a^	62.33	7.02	58.67	5.34	0.40
	Nano-Cu	4.16	1.37	2.79 ^a^	91.00 ^c^	11.51 ^b^	57.33	5.58	62.67	5.49	0.35
	Pb+Nano-Cu	4.13	1.56	2.57 ^ab^	103.00 ^ab^	12.47 ^b^	56.67	6.39	58.33	5.51	0.35
S.E.M.		0.20	0.25	0.18	2.19	0.64	3.70	0.50	2.77	0.52	0.06
*P*-Value	Species	0.8292	0.9662	0.8676	0.0520	0.1413	0.2942	0.8021	0.7377	0.7462	0.6984
	Treatment	0.0005	0.9761	0.0003	0.0000	0.0000	0.7793	0.0064	0.7806	0.9829	0.9448
	Interaction	0.6497	0.9821	0.6582	0.8055	0.4797	0.8442	0.7520	0.3431	0.9928	0.8937

1 Total protein (g/ dL).

2 Albumin (g/ dL).

3 Globulin (g/ dL).

4 Glucose (mg/ dL).

5 Total cholesterol (mg/ dL).

6 Triglyceride (mg/ dL).

7 Alanine aminotransferase.

8 Aspartate aminotransferase. Different letters indicate significant differences within each species at *P* < 0.05 (uppercase letters ^A.B,C,…etc.^: *Oreochromis niloticus*; lowercase letters ^a,b,c,…etc.^: *Oreochromis aureus*).

### Innate immune responses

**Fig. 4:**
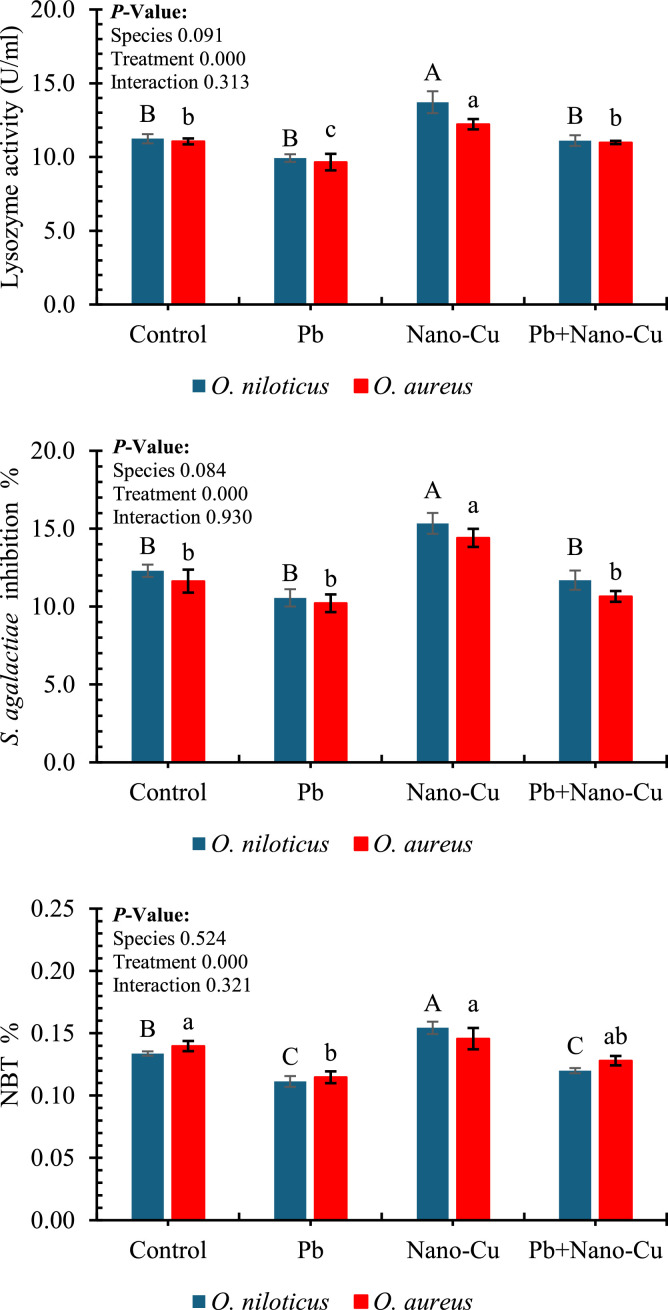
Innate immune responses in tilapias after 60 days of feeding trial. Different letters indicate significant differences within each species at *P* < 0.05 (uppercase letters ^A, B, C,…etc.^: *Oreochromis niloticus*; lowercase letters ^a, b, c,…etc.^: *Oreochromis aureus*).

Fig. 4 illustrates the effects of dietary lead (Pb), nano-copper (Nano-Cu), and their combination on key innate immune responses in *Oreochromis niloticus* and *Oreochromis aureus* after 60 days of feeding. Treatment had a highly significant effect (*p* < 0.001) on all measured immune indices: lysozyme activity, *S. agalactiae* inhibition %, and nitroblue tetrazolium (NBT) reduction, while species and interaction effects were not significant (*p* > 0.05). In terms of lysozyme activity, Pb exposure led to a significant reduction in both species (values around 10 U/mL), compared to the control and Nano-Cu groups. Fish fed the Nano-Cu diet showed the highest lysozyme activity (≈13.5–14 U/mL), which was significantly superior to all other treatments, reflecting enhanced non-specific immune defense. The Pb+Nano-Cu group displayed intermediate lysozyme levels, suggesting a partial recovery from Pb-induced immunosuppression. Similarly, *S. agalactiae* inhibition percentages followed the same pattern. Nano-Cu significantly improved the antibacterial capacity in both species (≈16–17 %), while the Pb group had the lowest inhibition values (≈12 %), indicating compromised immune function. Control and Pb+Nano-Cu groups showed moderate inhibition percentages, with the latter showing partial restoration of immune competence. Regarding NBT activity, which reflects respiratory burst activity of phagocytes, Nano-Cu supplementation again produced the highest responses (≈0.20–0.22 %), while Pb exposure significantly suppressed NBT values (≈0.15 %). The Pb+Nano-Cu combination resulted in partial improvement but remained significantly lower than the Nano-Cu group alone.

### Antioxidant enzyme and lipid peroxidation

**Fig. 5:**
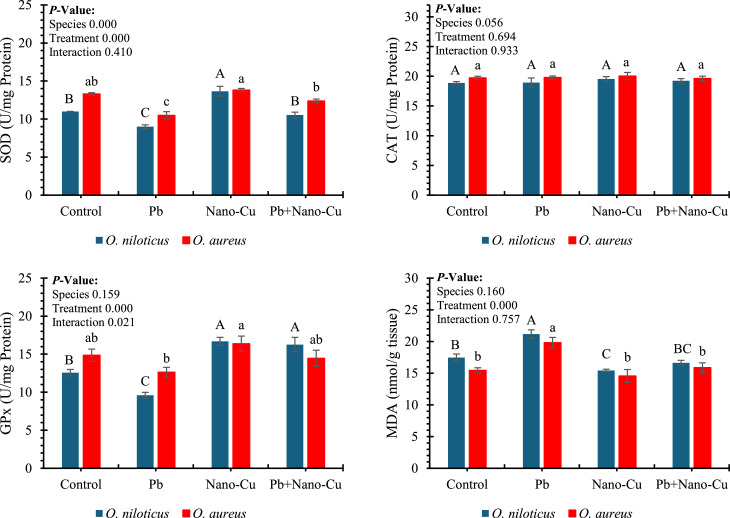
Antioxidant enzymes and lipid peroxidation in the livers of tilapias after a 60-day feeding period: SOD (superoxide dismutase), CAT (catalase), GSH-Px (glutathione peroxidase), and MDA (malondialdehyde). Different letters indicate significant differences within each species at *P* < 0.05 (uppercase letters ^A, B, C,…etc.^: *Oreochromis niloticus*; lowercase letters ^a, b, c,…etc.^: *Oreochromis aureus*).

Fig. 5 presents the effects of dietary lead (Pb), nano-copper (Nano-Cu), and their combination on hepatic antioxidant enzyme activities and lipid peroxidation in *Oreochromis niloticus* and *Oreochromis aureus* after a 60-day feeding trial. Treatment had a highly significant effect (*p* < 0.001) on all oxidative stress biomarkers, including superoxide dismutase (SOD), glutathione peroxidase (GPx), and malondialdehyde (MDA), whereas species and interaction effects were generally non-significant, except for a minor interaction in GPx (*p* = 0.021). Pb exposure significantly decreased antioxidant enzyme activities and increased MDA levels, indicating oxidative stress. In both species, Pb-fed groups exhibited the lowest SOD and GPx values, with *O. aureus* showing SOD as low as ∼13 U/mg and GPx around ∼17 U/mg protein. Conversely, Nano-Cu supplementation significantly enhanced SOD and GPx activities (≈19–20 U/mg protein), while Pb+Nano-Cu groups showed intermediate values, reflecting partial recovery from Pb-induced oxidative stress. MDA levels were highest in Pb-exposed fish (∼25 nmol/g tissue), whereas Nano-Cu reduced MDA significantly to ∼18–19 nmol/g, with partial mitigation also observed in the Pb+Nano-Cu group. Notably, catalase (CAT) activity was unaffected by treatment, species, or their interaction (*p* > 0.05).

### Gene expression

**Fig. 6:**
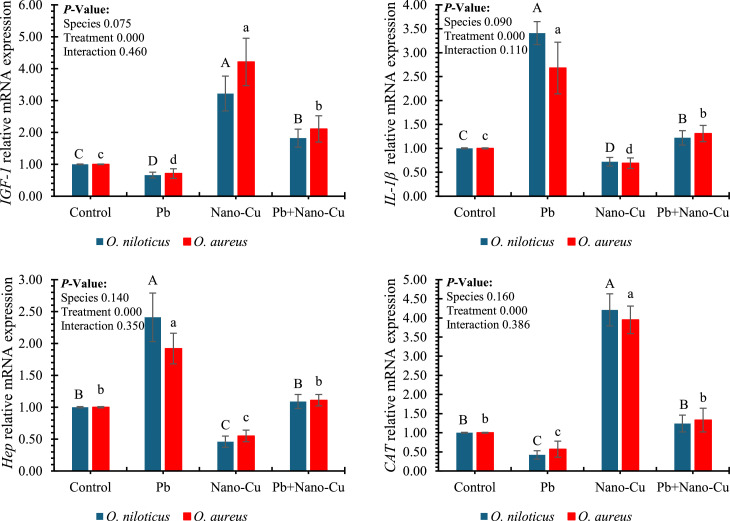
Effects of Nano-Cu supplementation on the hepatic expression of growth, immune, and antioxidant-related genes in tilapia after a 60-day feeding period. *IGF-1* (*insulin-like Growth Factor 1*), *IL-1 β* (*interleukin-1 Beta*), *Hep* (*hepcidin*), and *CAT* (catalase). Different letters indicate significant differences within each species at *P* < 0.05 (uppercase letters ^A, B, C,…etc.^: *Oreochromis niloticus*; lowercase letters ^a, b, c,…etc.^: *Oreochromis aureus*).

Fig. 6 illustrates the impact of Nano-Cu, Pb, and their interaction on hepatic gene expression of growth *(IGF-1*), immune *(IL-1β* and *Hepcidin*), and antioxidant-related (*CAT*) markers in tilapia following a 60-day feeding period. Treatment had a highly significant effect (*p* < 0.001) on all four genes, while neither species nor interaction effects were statistically significant (*p* > 0.05). *IGF-1* expression was significantly upregulated in Nano-Cu-fed fish (up to ∼5.0-fold in *O. niloticus*) compared to all other treatments, with Pb-fed fish showing the lowest expression (below 1-fold), indicating growth inhibition under heavy metal exposure. Co-supplementation with Nano-Cu and Pb moderately restored *IGF-1* levels. Similarly, *IL-1β* expression was drastically elevated in Pb-treated groups (≈3.5–4.0-fold), suggesting an inflammatory response, while Nano-Cu suppressed this expression significantly, indicating anti-inflammatory potential. Hepcidin (*Hep*) expression followed the opposite trend: strongly upregulated in Pb-treated fish (≈2.5-fold), and significantly downregulated in Nano-Cu treatments (≈1-fold), showing the modulatory role of Nano-Cu on iron regulation and immune signaling. For *CAT* gene expression, the pattern mirrored that of *IGF-1*, with Nano-Cu-fed groups exhibiting the highest expression (∼4.5-fold), suggesting antioxidant gene activation. In contrast, Pb treatment significantly downregulated *CAT* expression, which was only partially restored by co-supplementation.

## Discussion

Heavy metal contamination, particularly lead (Pb), poses significant risks to aquatic organisms, inducing oxidative stress, impairing growth, and disrupting immune function [25,45]. In fish, chronic Pb exposure has been linked to bioaccumulation in tissues, causing histopathological alterations and metabolic disturbances [26,46,47]. Copper (Cu), an essential trace element, plays a crucial role in numerous physiological processes including antioxidant defense and immune modulation [19,48]. However, excessive copper can be toxic, highlighting the importance of dose and bioavailability [11,49]. Nano-formulations of copper (Nano-Cu) have emerged as promising dietary supplements due to their enhanced bioavailability and potential to mitigate heavy metal toxicity [50,51]. This study builds on existing knowledge by exploring the protective effects of dietary nano-copper against Pb-induced toxicity in *O. niloticus* and *O. aureus*, with a focus on growth, immunity, and gene expression.

Growth performance reflects overall fish health, nutrient utilization, and the impact of toxicants or supplements on metabolic efficiency [12,52]. The significant reductions in BW_60_, WG %, SGR, and elevation of FCR in Pb-exposed groups align with previous findings that chronic Pb exposure impairs feed intake, nutrient assimilation, and metabolic function in fish [29,53]. Pb toxicity is known to disrupt mitochondrial integrity, suppress protein synthesis, and induce oxidative damage, all of which contribute to reduced anabolic processes and energy efficiency [26,54]. In contrast, dietary Nano-Cu significantly enhanced all growth parameters in both species, producing the highest BW_60_, WG %, and SGR, along with improved FCR values. This effect may be attributed to the critical biological roles of copper, particularly in enzymatic systems involved in energy metabolism and oxidative stress defense [2,11,16]. As a cofactor for enzymes such as cytochrome c oxidase and Cu/Zn-superoxide dismutase (SOD), copper is essential for ATP production and reactive oxygen species (ROS) detoxification [55]. Nano-formulated Cu offers enhanced bioavailability, facilitating more efficient cellular uptake and utilization compared to conventional copper salts [2,[56], [57], [58]]. The observed growth enhancement is consistent with previous studies reporting that low-dose Nano-Cu improves feed efficiency, appetite, and metabolic activity in various aquaculture species, including tilapia [2], Asian walking catfish [56] and red sea bream [16].

Interestingly, the group co-exposed to Pb and Nano-Cu demonstrated partially restored growth parameters, with values intermediate between the Pb and Nano-Cu-only groups. This finding indicates that Nano-Cu can counteract, at least in part, the deleterious effects of Pb exposure [59,60]. The protective mechanism may involve Nano-Cu’s ability to upregulate antioxidant defenses, support mitochondrial function, and enhance nutrient digestion and assimilation, thereby mitigating the metabolic disturbances induced by Pb [61,62]. Nevertheless, the partial nature of this amelioration suggests that while Nano-Cu reduces the impact of Pb toxicity, it does not completely eliminate the adverse effects, possibly due to persistent Pb accumulation or irreversible cellular damage.

Digestive enzymes indicate digestive capacity and nutrient absorption efficiency; affected by gut health and metal toxicity [63,64]. In terms of digestive enzyme activities, the data further substantiate the role of Nano-Cu in promoting intestinal health and digestive function. Amylase, protease, and lipase activities were significantly enhanced in Nano-Cu-supplemented fish, while Pb exposure markedly suppressed these enzymes, reflecting impaired digestive capacity. These enzymes are critical for the digestion of carbohydrates, proteins, and lipids, respectively, and their activity levels are reliable indicators of intestinal functionality and nutrient absorption efficiency [[65], [66], [67]]. The improved enzyme activity in Nano-Cu groups likely contributes to the enhanced growth performance observed, by supporting efficient nutrient utilization. These results are consistent with reports that trace minerals like Cu modulate pancreatic and brush border enzyme synthesis and secretion [1,68,69].

The species-specific variation observed, particularly in lipase activity, is noteworthy. *O. aureus* generally exhibited higher lipase activity than *O. niloticus*, especially under Nano-Cu and control diets. This difference may be attributed to inherent physiological or genetic traits, possibly linked to lipid metabolism efficiency or adaptive responses to dietary components. The significant interaction between species and treatment further supports the idea that species respond differently to mineral supplementation under stress conditions, which should be considered when formulating species-specific nutritional interventions [70,71].

Nano-Cu also effectively reversed Pb-induced suppression of digestive enzymes, further confirming its protective role. This may occur through restoration of intestinal epithelial integrity, reduction of oxidative stress, and stabilization of enzyme-producing cells, thereby sustaining normal digestive physiology under toxic conditions [62,64]. The ability of Nano-Cu to support enzymatic function under Pb exposure reinforces its potential as a nutritional intervention against environmental contaminants in aquaculture.

Histology reveals cellular and tissue-level damage or protection in organs (e.g., liver, intestine), validating physiological and biochemical findings [72]. The histopathological and bioaccumulation results of this study clearly demonstrate the detrimental effects of dietary Pb exposure on hepatic and intestinal tissues, as well as muscle lead accumulation in *O. niloticus* and *O. aureus*. Conversely, dietary supplementation with nano-copper (Nano-Cu) exhibited notable ameliorative effects, mitigating tissue damage and reducing lead deposition.

Histological alterations in the intestine were most pronounced in fish fed Pb-contaminated diets, with evident villus shortening, epithelial sloughing, and overall mucosal disintegration. These findings are consistent with earlier reports in teleosts, where Pb exposure has been shown to compromise gut morphology through oxidative damage, tight junction disruption, and inflammatory infiltration [29,73,74]. Pb's interference with calcium homeostasis and structural proteins impairs epithelial cell integrity, leading to compromised barrier function and reduced nutrient absorption [25,75]. Conversely, the Nano-Cu-supplemented group exhibited normal villus architecture and even mild enhancement in mucosal integrity, suggesting trophic effects of copper on intestinal epithelium. This aligns with findings from El-Erian, et al. [2], who reported that dietary Nano-Cu improved intestinal morphology in Nile tilapia, potentially by stimulating enterocyte proliferation and modulating antioxidant defenses at the mucosal level.

Importantly, co-exposure to Pb and Nano-Cu led to partial restoration of intestinal structure, with improved villus height and epithelial organization compared to the Pb-only group. This suggests that Nano-Cu confers a protective effect against Pb-induced intestinal damage, likely through attenuation of oxidative stress and inflammation. Copper nanoparticles are known to enhance antioxidant enzyme activity, reduce lipid peroxidation, and support cellular repair processes [[76], [77], [78]]. Furthermore, their nanoscale size facilitates targeted cellular interactions, improving bioavailability and functional efficacy in epithelial tissues.

The liver histology similarly revealed the hepatotoxic nature of Pb and the protective properties of Nano-Cu. Pb-exposed fish showed characteristic signs of hepatic damage, including hepatocyte vacuolization, sinusoidal dilation, and leukocytic infiltration, indicative of oxidative injury, mitochondrial dysfunction, and immune activation [79,80]. These histological lesions correlate well with earlier studies where Pb exposure triggered hepatic necrosis, inflammation, and metabolic dysregulation in fish [26,27,81]. In contrast, Nano-Cu supplementation maintained near-normal hepatic architecture, with only minor vacuolization, reinforcing its biocompatibility and potential hepatoprotective effect at the administered dosage. The marked structural recovery observed in the liver and pancreas of Pb+Nano-Cu co-treated fish suggests that Nano-Cu can mitigate Pb-induced hepatocellular damage. This protective role may be mechanistically attributed to the upregulation of copper-dependent antioxidant enzymes such as Cu/Zn-SOD, which neutralize ROS and stabilize hepatocyte membranes [1,3]. Furthermore, copper’s role in mitochondrial electron transport and redox regulation may counteract the mitochondrial disruption induced by Pb toxicity [54,82,83].

Muscle lead accumulation data further validate the protective role of Nano-Cu. As expected, fish fed Pb-contaminated diets exhibited the highest concentrations of lead in muscle tissue, exceeding 2.8 mg/kg and 3.4 mg/kg in *O. niloticus* and *O. aureus*, respectively. These levels reflect significant Pb bioavailability and tissue retention, which not only pose physiological risks to fish but also raise public health concerns regarding food safety. Chronic Pb accumulation in muscle can interfere with muscle protein function and contractility, as well as introduce Pb into the human food chain [84,85]. Notably, dietary Nano-Cu significantly reduced muscle Pb concentrations in co-treated fish, indicating a chelation or sequestration effect that limits Pb uptake or enhances its excretion. Although Pb levels in the Pb+Nano-Cu group remained higher than controls, the marked reduction compared to the Pb-only group suggests an important ameliorative mechanism. This may involve competitive absorption between Cu and Pb at intestinal transporters such as divalent metal transporter 1 (DMT1), or induction of metal-binding proteins like metallothioneins that preferentially bind toxic metals [86,87]. These results echo previous findings in Asian walking catfish and carp, where Nano-Cu reduced tissue accumulation of heavy metals and mitigated their toxic effects [56,88].

Taken together, Pb exposure induces severe histopathological lesions in both intestinal and hepatic tissues and results in significant Pb accumulation in muscle tissue, adversely affecting fish health and food safety. Nano-Cu supplementation, however, exerts a dual role: promoting tissue integrity and growth under normal conditions and conferring substantial protection under Pb-induced stress. The underlying mechanisms involve enhanced antioxidant defense, reduced intestinal Pb uptake, improved tissue regeneration, and stabilization of cellular structure. These findings support the potential application of nano-Cu as a dietary additive in aquaculture for mitigating the toxicity of environmental pollutants.

The current study offers a holistic evaluation of the physiological and molecular impacts of dietary Pb, Nano-Cu, and their interaction on *O. niloticus* and *O. aureus*. Over 60 days, clear and consistent trends were observed across multiple parameters, indicating that Pb exposure induces severe systemic stress, while Nano-Cu supplementation offers substantial protective and modulatory effects. Serum biochemistry provides insight into metabolic, hepatic, renal, and stress status; includes glucose, proteins, liver enzymes, and lipids [89,90]. Pb exposure significantly elevated serum glucose levels in both tilapia species, consistent with previous studies that report hyperglycemia as a hallmark of environmental stress [81]. Elevated glucose likely results from hypothalamic–pituitary–interrenal (HPI) axis activation, leading to enhanced glucocorticoid release and hepatic gluconeogenesis. Conversely, Nano-Cu supplementation significantly reduced glucose concentrations, reflecting its stress-mitigating effects, likely mediated through improved cellular homeostasis and suppression of stress-related signaling pathways [24,91].

Regarding the protein profile, Pb exposure caused a reduction in total protein and globulin levels, indicative of impaired hepatic protein synthesis and compromised immune function. This is in agreement with the findings of Zahran, et al. [92], who linked heavy metal-induced hepatotoxicity to reduced synthesis of immune-relevant plasma proteins. In contrast, Nano-Cu significantly enhanced these indices, particularly globulin levels, suggesting an immunostimulatory role. This may be attributed to the improved nutrient utilization and the role of Cu as a cofactor in enzymatic processes involved in protein synthesis [2,93].

Total cholesterol (TChol) levels were significantly reduced in fish exposed to Nano-Cu and Pb+Nano-Cu compared to the control and Pb-only groups. While this observation was initially interpreted as a possible hypocholesterolemic effect of Nano-Cu, such a reduction may also reflect metabolic disturbances rather than a purely beneficial outcome [2]. Lead exposure is known to disrupt lipid metabolism by impairing hepatic function, inhibiting key enzymes in cholesterol biosynthesis, and reducing food intake, all of which can result in lower circulating cholesterol levels [94,95]. Similarly, excessive exposure to metallic nanoparticles can induce oxidative stress and alter hepatic lipid homeostasis, leading to decreased serum cholesterol [11,96]. Therefore, the decline in cholesterol observed in Nano-Cu and Pb+Nano-Cu groups may partly stem from Pb-induced metabolic stress or nanoparticle-related modulation of hepatic lipid metabolism, rather than a direct, beneficial cholesterol-lowering effect. This interpretation is supported by previous findings where metal exposure reduced plasma lipids through hepatocellular damage and altered enzymatic activity [2,97]. Consistent with this, Pb exposure significantly elevated serum ALT activity, confirming hepatocellular injury and leakage of intracellular enzymes into the bloodstream, a hallmark of hepatic dysfunction [89,98]. Interestingly, fish treated with Nano-Cu maintained ALT levels close to the control, suggesting that at the tested concentration, Nano-Cu may exert a partial hepatoprotective effect, likely through enhancement of antioxidant defenses and stabilization of cell membranes [99].

Innate immunity assesses non-specific defense mechanisms (e.g., lysozyme, NBT); important for resistance against pathogens and immune competence [100]. All measured innate immune indices, lysozyme activity, *S. agalactiae* inhibition, and nitroblue tetrazolium (NBT) reduction, were significantly influenced by treatment. Pb exposure significantly reduced these indices, confirming its immunosuppressive nature, likely mediated by oxidative stress-induced leukocyte dysfunction and cytokine dysregulation [74,101,102]. Conversely, Nano-Cu supplementation significantly enhanced innate immune responses, suggesting immune activation and potential immunopotentiation. However, it should be noted that increases in lysozyme and NBT activity under Pb exposure could also reflect immune activation or inflammation, which may not necessarily indicate a healthy immune status. The role of Nano-Cu in immune enhancement should be considered in combination with other relevant immune indicators, such as cytokine expression, to provide a more comprehensive understanding of its immunomodulatory effects. This effect may be attributed to macrophage activation, enhanced phagocytosis, and modulation of cytokine expression [16,78,103]. The partial recovery of physiological and biochemical parameters in the Pb + Nano-Cu group indicates that Nano-Cu supplementation markedly alleviated but did not completely reverse Pb-induced stress. This outcome likely reflects multiple interacting mechanisms. First, Pb causes oxidative and mitochondrial damage that can be irreversible or only slowly reversible within the experimental period, particularly in hepatic and renal tissues where metal accumulation is greatest. Second, although the dietary Nano-Cu dose (2 mg kg⁻¹) was selected for safety and efficacy, it may represent a suboptimal protective level against severe Pb exposure, as higher concentrations could further support antioxidant and metallothionein responses—but must be balanced against Cu toxicity risks. Third, competitive absorption dynamics between Pb and Cu at the intestinal level (via DMT1 and other metal transporters) may limit Cu uptake and bioavailability under Pb stress, thereby constraining Nano-Cu’s systemic effectiveness. Finally, residual Pb burden in tissues can sustain low-grade oxidative and inflammatory signaling, preventing complete biochemical normalization. These factors together explain why Nano-Cu achieved significant mitigation but not total recovery of physiological homeostasis under Pb challenge.

Antioxidant status measures oxidative stress and defense (e.g., SOD, GPx, MDA); key for evaluating redox balance under toxicity or supplementation [104]. The oxidative stress biomarkers, SOD, GPx, and MDA, responded significantly to treatments, reinforcing the central role of redox balance in mediating toxicological and protective responses [105]. Pb exposure suppressed SOD and GPx activities and elevated MDA, indicative of excessive ROS production, lipid peroxidation, and impaired antioxidant defenses [102,106]. These oxidative insults can impair cellular function, damage macromolecules, and trigger inflammation. Nano-Cu supplementation restored antioxidant enzyme activities and reduced MDA levels, reflecting a strengthened antioxidant defense system. The improved redox status may be attributed to Cu’s role in the active sites of Cu/Zn-SOD and in catalyzing redox reactions essential for ROS detoxification [1,103]. Interestingly, CAT activity remained unaltered, suggesting enzyme-specific responses to the dietary treatments.

Gene expression markers provide critical mechanistic insights into the molecular responses elicited by toxicants (e.g., Pb) and mitigators (e.g., Nano-Cu), particularly in pathways related to growth (*IGF-1*), inflammation (*IL-1β*), immunity (*hepcidin*), and oxidative stress (*CAT*), thereby elucidating their functional impacts on fish physiology [107,108]. The gene expression data provided further mechanistic insight into the physiological modulation induced by Pb and Nano-Cu. Pb exposure downregulated *IGF-1*, a growth-related gene, and *CAT*, an antioxidant gene, while upregulating *IL-1β* and *hepcidin*, both associated with inflammatory and immune responses. These alterations confirm that Pb exerts multi-level biological stress, impairing growth, oxidative defense, and immune equilibrium [109]. The upregulation of *IL-1β* and *hepcidin* implies an inflammatory hepatic response and iron dysregulation, hallmarks of metal-induced hepatopathy. In contrast, Nano-Cu significantly enhanced *IGF-1* and *CAT* expression, while suppressing *IL-1β* and *hepcidin*, indicating an orchestrated enhancement of growth, antioxidant, and anti-inflammatory capacities. *IGF-1* upregulation likely reflects improved nutritional status and liver function, while enhanced *CAT* transcription aligns with observed enzymatic activity. The downregulation of *IL-1β* and hepcidin may result from reduced tissue inflammation and oxidative injury, affirming Nano-Cu’s immunomodulatory and hepatoprotective roles [1,11].

Collectively, these results demonstrate that Pb exerts toxicity through oxidative stress, immune suppression, inflammation, and hepatic dysfunction. Nano-Cu mitigates these effects via enhancement of antioxidant defenses, stabilization of key gene expressions, and activation of innate immunity. The consistency of responses across *O. niloticus* and *O. aureus* suggests the broad applicability of Nano-Cu supplementation in tilapia culture. However, the intermediate recovery observed in Pb+Nano-Cu groups indicates that while Nano-Cu significantly alleviates Pb toxicity, it does not fully neutralize its adverse effects.

These findings reinforce previous evidence on the protective role of trace elements and nanonutrients in aquaculture and underscore Nano-Cu’s potential as a functional dietary additive for managing environmental metal stress. Nevertheless, the present work primarily focused on functional innate immune assays (lysozyme, bactericidal activity, and NBT reduction), which provide valuable indicators of non-specific defense capacity but do not encompass the full complexity of immune regulation. Future studies will extend immunological profiling to include quantitative assessment of humoral and cellular markers such as complement component C3, immunoglobulin M (IgM), and cytokines (e.g., TNF-α, IL-10) to better elucidate the mechanistic basis of Nano-Cu–mediated immune modulation under Pb-induced stress.

## Conclusion

The present study clearly demonstrates that dietary supplementation with nano-copper exerts a significant protective effect against lead-induced toxicity in both *Oreochromis niloticus* and *O. aureus*. Nano-Cu enhanced growth performance, improved digestive enzyme activity, and restored serum biochemical balance. It also boosted antioxidant defenses and downregulated markers of oxidative stress and inflammation at the molecular level. Although co-administration with lead did not fully restore all physiological parameters to control levels, it substantially mitigated the adverse effects compared to lead exposure alone. These findings suggest that Nano-Cu holds promise as a functional dietary additive for reducing the health risks of heavy metal exposure in aquaculture, with consistent benefits observed across different tilapia species. Future studies should focus on expanding immunological profiling by assessing cytokine expression and the quantification of major immune proteins (C3, IgM) to provide deeper insight into Nano-Cu’s role in modulating innate and adaptive immunity. Additionally, long-term investigations under various environmental stressors are warranted to confirm the sustainability and safety of these effects.

## Funding

None.

## CRediT authorship contribution statement

**Mohammed F. El Basuini:** Conceptualization, Methodology, Software, Validation, Formal analysis, Investigation, Resources, Data curation, Visualization, Supervision, Project administration, Funding acquisition, Writing – original draft, Writing – review & editing. **Rawheya Shaaban Ramadan:** Methodology, Formal analysis, Investigation, Visualization. **Medhat E. Eldenary:** Methodology, Validation, Formal analysis, Investigation, Resources, Data curation. **Islam I. Teiba:** Conceptualization, Methodology, Software, Validation, Formal analysis, Investigation, Resources, Data curation, Visualization, Writing – original draft, Writing – review & editing. **Ali A. Soliman:** Methodology, Investigation, Resources, Data curation, Visualization. **Mahmoud S. Gewaily:** Methodology, Data curation, Writing – review & editing. **Issam Khelfaoui:** Methodology, Software, Investigation, Resources, Data curation, Visualization. **Mayada Alhoshy:** Methodology, Software, Validation, Formal analysis, Investigation, Resources, Data curation, Visualization. **Akram Ismael Shehata:** Conceptualization, Methodology, Software, Validation, Formal analysis, Investigation, Resources, Data curation, Visualization, Supervision, Project administration, Funding acquisition, Writing – original draft, Writing – review & editing.

## Declaration of competing interest

The authors declare no conflict of interest.

## Data Availability

Data will be made available on request.
